# Seasonal evolution of *Drosophila melanogaster* abdominal pigmentation is associated with a multifarious selective landscape

**DOI:** 10.1093/evolut/qpag038

**Published:** 2026-03-11

**Authors:** Skyler Berardi, Jack K Beltz, Seth M Rudman, Tess N Grainger, Jonathan M Levine, Hayes Oken, Paul Schmidt

**Affiliations:** Department of Biology, University of Pennsylvania, Philadelphia, PA 19104, United States; Department of Ecology and Evolutionary Biology, Princeton University, Princeton, NJ 08544, United States; Department of Biology, University of Pennsylvania, Philadelphia, PA 19104, United States; Department of Biology, University of Pennsylvania, Philadelphia, PA 19104, United States; School of Biological Sciences, Washington State University, Vancouver, WA 98686, United States; Department of Ecology and Evolutionary Biology, Princeton University, Princeton, NJ 08544, United States; Department of Integrative Biology, University of Guelph, Guelph, ON, N1G 2W1, Canada; Department of Ecology and Evolutionary Biology, Princeton University, Princeton, NJ 08544, United States; Department of Biology, University of Pennsylvania, Philadelphia, PA 19104, United States; Department of Biology, University of Pennsylvania, Philadelphia, PA 19104, United States

**Keywords:** adaptation, trait evolution, experimental evolution

## Abstract

Pigmentation has been widely studied by evolutionary biologists due to both ease of measurement and relationship to fitness. *Drosophila melanogaster* pigmentation has represented a particularly useful avenue of investigation, as extensive genetic tools have enabled the characterization of the trait’s complex architecture. *Drosophila* pigmentation also varies predictably across space and time in wild populations, suggesting pigmentation is a component of adaptation to local environmental conditions. Despite this, the impact of *D. melanogaster* pigmentation on fitness, and the environmental factors that drive the evolution of pigmentation, are not well understood. To address this gap, we experimentally evolved replicated *D. melanogaster* populations in field mesocosms to determine whether and how pigmentation evolves in response to environmental variation. We found that pigmentation rapidly and predictably adapted to a direct manipulation of temperature, supportive of melanization playing a role in thermoregulation. However, we also determined that pigmentation responded adaptively to direct manipulations of numerous additional factors, including intraspecific competition, diet, and the microbiome. These findings suggest that the selective landscape acting on pigmentation is complex and multifaceted, and that patterns of melanization may be driven, at least in part, by indirect selection due to correlations with other fitness-related traits.

## Teaser

Despite *Drosophila melanogaster* pigmentation being a well-studied phenotype, the fitness benefits of cuticle melanization in wild populations remain obscured. We experimentally evolved fly populations in response to manipulated environmental parameters to define the selective landscape of pigmentation. We first tested whether temperature drives adaptive shifts, and we found that the genetic response to temperature aligned with predictions of thermal melanism during the summer, but not the fall. However, during the fall period, patterns of phenotypic plasticity were concordant with thermal melanism. Interestingly, several additional environmental manipulations altered pigmentation patterns, indicating that the fitness landscape for pigmentation is highly multifaceted.

## Introduction

Pigmentation represents a well-studied phenotype across a breadth of taxa, and it is a particularly useful model for defining how selective pressures shape dynamics of adaptation. Patterns of coloration can be characterized visually, and links between genotype and phenotype have been established across wild populations of numerous species (Hoekstra, 2006; Kronforst et al., 2012). The adaptive significance of pigmentation is straightforward in many systems, yielding clear predictions for how environmental change will interact with coloration patterns. Adaptive pigmentation shifts are often driven by crypsis, with notable examples including melanism in the peppered moth (Cook et al., 2012) and pygmy grasshopper (Forsman et al., 2011), blanched coloration in White Sands lizards (Rosenblum et al., 2004), coat color shifts in mice (Barrett et al., 2019), and seasonal camouflage in snowshoe hares (Jones et al., 2018; Zimova et al., 2016). Alternatively, pigmentation patterns can be shaped by thermoregulation (Kingsolver, 1987; Watt, 1968), UV tolerance (Jablonski & Chaplin, 2010), or aposematism (Mappes et al., 2005). However, the connection between pigmentation and fitness is less clear for other species, and this includes the model fly *Drosophila melanogaster*.

Despite *D. melanogaster* pigmentation being an established model for studying the genetic basis of complex trait development (Massey & Wittkopp, 2016; Wittkopp et al., 2003), we have yet to unambiguously identify the agents of selection that shape observed variance in melanization. *Drosophila* pigmentation exhibits patterns suggestive of adaptation in wild populations: parallel clines in melanization have repeatedly emerged across latitudinal and altitudinal gradients on multiple continents, in which flies are consistently darker at higher latitudes and altitudes (Bastide et al., 2014; Berardi et al., 2025; Das, 1995; David et al., 1985; Dev et al., 2013; Munjal et al., 1997; Parkash et al., 2008; Pool & Aquadro, 2007; Rajpurohit et al., 2008a; Telonis-Scott et al., 2011). These clinal patterns are concordant with the thermal melanism hypothesis, which predicts that darker pigmentation will be favored under colder conditions as a mechanism for elevating body temperature, and lighter pigmentation will be favored in warmer environments to mitigate overheating (Clusella-Trullas et al., 2007; Gibert et al., 2000; Gibson & Falls, 1979; Goulson, 1994; Kingsolver & Wiernasz, 1991; Watt, 1969). While it was previously thought that *D. melanogaster* are too small to thermoregulate via pigmentation (Willmer & Unwin, 1981), darker cuticle pigmentation can be associated with a higher body temperature when flies are exposed to light (Freoa et al., 2023). Additionally, *D. melanogaster* pigmentation is developmentally plastic with respect to temperature, where elevated temperature decreases melanization and lowered temperature increases melanization (David et al., 1990; Gibert et al., 2007; Lafuente et al., 2024). Therefore, several lines of evidence suggest that *D. melanogaster* melanization may directly support thermoregulation, similar to various insect and reptile species (Clusella-Trullas et al., 2007).

However, the link between temperature and *Drosophila* pigmentation remains inconclusive. We recently demonstrated that *D. melanogaster* pigmentation evolves rapidly and cyclically across seasons in temperate populations, in which individuals evolve to become darker over the winter and lighter in the summer (Berardi et al., 2025). While these patterns broadly tracked with predictions of thermal melanism, we did find an inconsistency: flies remained genetically light late into the fall, despite decreases in daily temperatures (Berardi et al., 2025). This indicates that temperature may not be the sole agent of selection driving melanization patterns, and several additional hypotheses have been proposed to explain the fitness relevance of *D. melanogaster* pigmentation. Increased melanization provides enhanced UV tolerance in *Drosophila* (Bastide et al., 2014), similar to humans (Jablonski & Chaplin, 2010). Alternatively, melanization may yield structural benefits associated with cuticular strength (Kronforst et al., 2012), and increased melanization correlates with improved desiccation tolerance (Rajpurohit et al., 2008b) and immune responses (Nakhleh et al., 2017; Subasi et al., 2024). The canonical *D. melanogaster* pigmentation genes are also highly pleiotropic (Wittkopp & Beldade, 2009); thus, selective pressures driving change in correlated traits may indirectly shift pigmentation patterns (Rajpurohit et al., 2016). Finally, prior investigation has demonstrated that insect pigmentation can be metabolically costly to produce in resource-limited environments (Ethier et al., 2015; Roff & Fairbairn, 2013), so variation in resource availability and competition may modulate *Drosophila* melanization. Thus, while temperature remains a putative driver of pigmentation patterns, we can hypothesize that additional agents of selection may directly, or indirectly, impact the seasonal evolution of this complex trait.

A powerful way to test these hypotheses is to manipulate putative environmental drivers of *D. melanogaster* melanization and measure evolutionary trajectories of pigmentation. Our finding that pigmentation evolves seasonally (Berardi et al., 2025) presented us with the opportunity to directly associate rapid evolutionary change in pigmentation with specific environmental shifts. Thus, to define the selective landscape of *D. melanogaster* pigmentation, we experimentally evolved replicated fly populations across seasons in field mesocosms and manipulated several abiotic and biotic conditions. Our experimental system enabled us to use parallelism across independent replicates to identify adaptive divergence between treatments (Schluter & Nagel, 1995) and to eliminate effects of cryptic structure and/or migration in affecting rapid evolution of pigmentation phenotype. We first tested the hypothesis that temperature drives *D. melanogaster* melanization by artificially increasing the temperature in field mesocosms and assessing whether populations adapted to be less melanized, as predicted by thermal melanism. We then manipulated four additional abiotic and biotic factors that have known associations with seasonal evolution in *Drosophila*: intraspecific competition (Bitter et al., 2026), interspecific competition (Grainger et al., 2021), diet (Beltz et al., 2025), and the gut microbiome (Rudman et al., 2019). Our suite of environmental manipulations allowed us to test whether shifts in melanization appear to be driven solely by temperature to produce a clear fitness benefit (i.e., thermoregulation), or alternatively, if numerous seasonally cycling conditions influence evolutionary patterns. We would predict the latter case to be true if pigmentation is under frequent indirect selection due to correlations with other fitness-related traits that evolve seasonally, yielding a multidimensional selective landscape.

## Materials and methods

### Experimental orchard system

To directly test hypotheses regarding the selective landscape of *D. melanogaster* pigmentation, we designed a series of experiments to examine the effects of targeted environmental manipulations on pigmentation shifts. We experimentally evolved replicated *D. melanogaster* populations in field mesocosms (8 m^3^ mesh cages) located in Philadelphia, PA, according to methodology described by: Rajpurohit et al. (2017), Rajpurohit et al. (2018), Rudman et al. (2019), Grainger et al. (2021), Rudman et al. (2022), Bitter et al. (2024), Beltz et al. (2025), Berardi et al. (2025), Karageorgi et al. (2025). The pigmentation data detailed in this paper were compiled across 4 years (2017, 2019, 2020, 2022). In each experiment year, we founded field mesocosms with a replicated, outbred *D. melanogaster* baseline population; notably, a different baseline population was used in each year (detailed below). This enabled us to measure parallel shifts in pigmentation from early summer to late fall and determine whether manipulated populations consistently exhibited a differential response relative to controls. By identifying parallel shifts in melanization across replicate populations, and by using our mesocosm system to eliminate confounding effects of migration and demography, we can conclude that observed shifts are most likely driven by adaptation.

Population density was reduced to lessen intraspecific competition in two years of experimentation: 2017 and 2022. In 2017, 150 isofemale lines originally collected from wild orchard populations in southeastern Pennsylvania were recombined to create an outbred baseline population (described in Rudman et al., 2025). We founded our field cages with this starting population on June 15. Eight replicate cages were subjected to control conditions (no manipulation, but seasonally evolving), and in an additional eight cages the population density was reduced by removing ~15% of embryos on the food supply/oviposition substrate every 2 days. Across four timepoints (August 3, September 22, October 18, and November 12), >1,000 eggs were collected from each cage and brought into the laboratory for two generations of common garden rearing (Rudman et al., 2025). Previous work has demonstrated that this collection and rearing protocol is sufficient to preclude effects of drift due to subsampling (Beltz et al., 2025; Bitter et al., 2024; Karageorgi et al., 2025; Rudman et al., 2022). F3 females were preserved 3–5 days post-eclosion in 75% EtOH at -20°C for later pigmentation scoring; samples of the baseline population for this experiment were lost and not scored.

We separately manipulated intraspecific competition and temperature in 2022. 76 inbred lines originally collected from Media, PA, were recombined across four generations to construct an outbred baseline population (as described in Bitter et al., 2024); this population was then used to found 27 replicate cages (each with 500 males and 500 females from a single 24 hr, density-controlled cohort) on July 6. We reduced population density to lessen intraspecific competition in 9 cages, we increased temperature in 9 cages, and 9 cages were unmanipulated controls. Across treatments, populations were provided a fresh loaf pan of standard cornmeal-molasses food every 2 days. To manipulate population density, we reduced the number of eggs laid on the food substrate every 2 days to 1,000, representing low-density development conditions based on the ratio of food volume to eggs (Shabalina et al., 1997). When unmanipulated, egg counts in control populations routinely exceeded 10,000 eggs per feeding (every 2 days). To manipulate temperature, we artificially warmed a subset of mesocosms by wrapping them in polyethylene greenhouse material to generate an open-top chamber effect (Welshofer et al., 2018). This passively increased the mean daily high temperature in warming cages by 0.99°C relative to control cages during the summer phase and by 0.73°C during the fall (Table S1). We collected > 1,000 eggs from each cage on September 7 and November 8 by letting populations lay eggs on fresh food substrate for 24 hr, brought the eggs into the lab for rearing, and preserved females for pigmentation scoring as described above following two generations of common garden, density-controlled treatment (25°C, 12L:12D).

In 2019, we manipulated interspecific competition between *D. melanogaster* and *Zaprionus indianus* as described in Grainger et al. (2021). This experiment was initiated on July 9 using an outbred founder population that was established by recombining 150 isofemale lines initially collected from wild orchard populations in southeastern Pennsylvania. Here, we focused on the subset of 14 seasonally evolving cages that either experienced interspecific competition (7 cages) or control conditions (7 cages). During the summer phase, *Z. indianus* individuals were introduced to treatment cages to impose interspecific competition. All flies were removed from the cages at the end of summer, and a subset of 5,000 *D. melanogaster* field-caught individuals was reintroduced to each cage to initiate the fall phase. We equalized abundances at the outset of the fall phase, rather than retaining cage-level differences in abundance that arose across the summer, to isolate how evolution in response to competition impacts adaptive trajectories from the ecological effects of competition (e.g., changes in abundance; see Grainger et al., 2021). Adult females corresponding to baseline, summer (September 11), and fall (November 8) timepoints were saved for pigmentation measurements following egg collection from each cage and common garden treatment (as described in the 2022 experiments). In addition to scoring pigmentation from common garden reared flies, we also collected females from each control cage by aspiration and preserved them immediately in 75% ethanol. We then scored these females to compare how pigmentation patterns differed between outdoor populations and common garden treated populations for each control cage.

The final two manipulations, diet and food supplementation with specific, gut-colonizing microbes, were conducted in 2020 (Beltz et al., 2025). Here, 80 inbred lines collected from southeastern Pennsylvania were recombined to generate an outbred founder population (as described in Rudman et al., 2022). On July 15, we seeded orchard cages with replicates of our founder population to investigate the effects of manipulated diet and microbiome. To test effects of food quality, we fed flies an apple-based diet containing less protein, fat, and carbohydrate content relative to standard cornmeal-molasses fly food, as described in Beltz et al. (2025). We then investigated the effects of manipulating the gut microbiome by consistently feeding flies a diet of apple food supplemented with one of two *D. melanogaster* resident microbes: *Acetobacter thailandicus* and *Lactobacillus brevis* (Pais et al., 2018). Treatments were randomly assigned to orchard cages: control food (*N* = 6 cages), apple food (*N* = 6 cages), apple food supplemented with *A. thailandicus* (*N* = 6 cages), and apple food supplemented with *L. brevis* (*N* = 6 cages). Over 1,000 eggs were collected from each cage across 5 timepoints, in addition to the baseline, for the diet treatment (August 13, September 7, September 30, November 9, and November 24), and across 3 timepoints for the microbial addition treatments (September 7, November 9, and November 24). Collected populations were subjected to two generations of laboratory, common garden treatment (25°C, 12L:12D) on the same culture medium they were fed in the field (i.e., control cages were reared on standard food, while diet and microbe treatments were reared on apple food). Because the microbes were added to apple food, the control for the microbial experiments is the apple treatment populations.

### Scoring abdominal pigmentation

Measurements of abdominal pigmentation were made using a rubric that assigns a score of 1–10 to each abdominal tergite based on % melanization (David et al., 1990), where a score of 10 corresponds to 100% melanization. Individual scores for each tergite are then summed to calculate an overall “pigmentation score” for the fly. Preserved females were scored during the same timeframe and blind to sample identity to avoid bias, and the first author quality checked all measurements. We only scored female abdominal pigmentation, as it is more variable than male abdominal pigmentation, and adult females were aged 3–5 days following eclosion to standardize development before scoring. For all experiment years besides 2017, only the three most distal tergites (tergites 5–7) were scored (David et al., 1990). In 2017 (manipulation of intraspecific competition), all 7 abdominal tergites were scored as in Rajpurohit et al. (2016).

We determined whether each treatment significantly impacted pigmentation by running a linear mixed effects model in R (RStudio v.2024.04.1 + 748) using the function “lmer” (“lme4,” v.1.1.35.5); significance was assessed with ANOVA (“lmerTest” v.3.1.3). For each experiment, timepoint, treatment, and their interaction were modeled as fixed effects, and cage was included as a random effect per the formula “lmer(Pigmentation Score ~ Timepoint * Treatment + (1|Cage).” For each model, we ran planned comparisons to test (i) differences between treatment and control pigmentation at the end of the summer and fall phases and (ii) significant shifts in pigmentation from summer to fall for each treatment. Here, we used the function “contrast” (“emmeans,” v.1.10.2) to run *t-*tests across each planned comparison; we have reported raw and Holm corrected *p-*values (Tables S2-S8). Data for each experiment was plotted using “ggplot2” (v.3.5.1), and R packages “tidyverse” (v.2.0.0), “readxl” (v.1.4.3), and “reshape2” (v.1.4.4) were used in data analysis. Figures were made using Keynote (v.8.1). Raw data and scripts associated with analyses for this project are available on GitHub.

## Results

### Testing the response of *D. melanogaster* pigmentation to seasonally changing temperature

**Figure 1.**
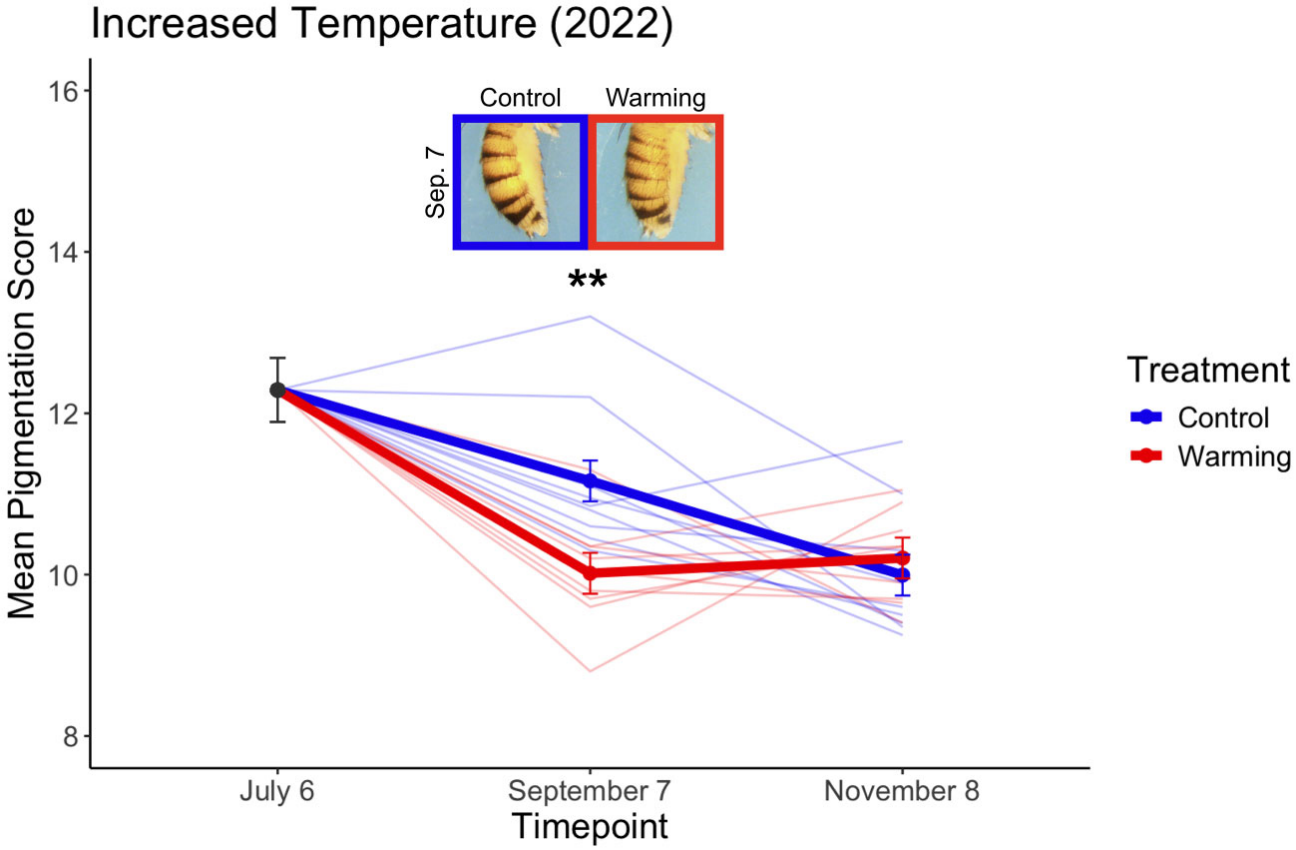
Increasing temperature reduces melanization during the summer phase. (A) Populations subjected to our “warming” treatment exhibited significantly lighter pigmentation relative to the control populations following the summer phase, but the populations did not differ in melanization following the fall phase of evolution. The mean pigmentation score across each treatment (± SE) is plotted alongside individual cage scores (thin lines). Representative images depicting the mean pigmentation score for the control and warming treatments are shown for the summer phase (**p < .01).

We experimentally evolved replicated *D. melanogaster* populations in field mesocosms across four years (2017, 2019, 2020, and 2022), and in each year, we manipulated environmental parameters and measured resulting effects on evolutionary trajectories of pigmentation. In 2022, we tested whether temperature is a significant driver of seasonal pigmentation patterns by increasing the mean daily high temperature by 0.73°C–0.99°C in warming populations (*N* = 9) relative to controls (*N* = 9; see *Materials and methods* section). We found increasing the temperature altered the adaptive trajectory of melanization over time compared to controls (Figure 1; Table S2A; *F* = 7.32, *p* = .007). Warming treatment populations exhibited significantly lighter pigmentation following the summer phase of seasonal evolution (Table S2B; *t* = 3.20, *p* = .007), and this result aligned with the prediction that reduced melanization would be favored in warmer temperatures to prevent overheating (Freoa et al., 2023). However, pigmentation patterns were not concordant with thermal melanism during the fall phase: as daily temperatures dropped, both warming and control populations would be predicted to evolve darker pigmentation, and the control populations to be the most melanized. Instead, we observed that control populations evolved to be genetically lighter in the fall, as seen in previous work (Berardi et al., 2025), and melanization did not significantly differ between treatments (Figure 1; Table S2B). Thus, our findings support the hypothesis that temperature is a key driver of adaptive pigmentation patterns during the summer, but not the fall.

### Modulation of *D. melanogaster* pigmentation by plasticity

**Figure 2.**
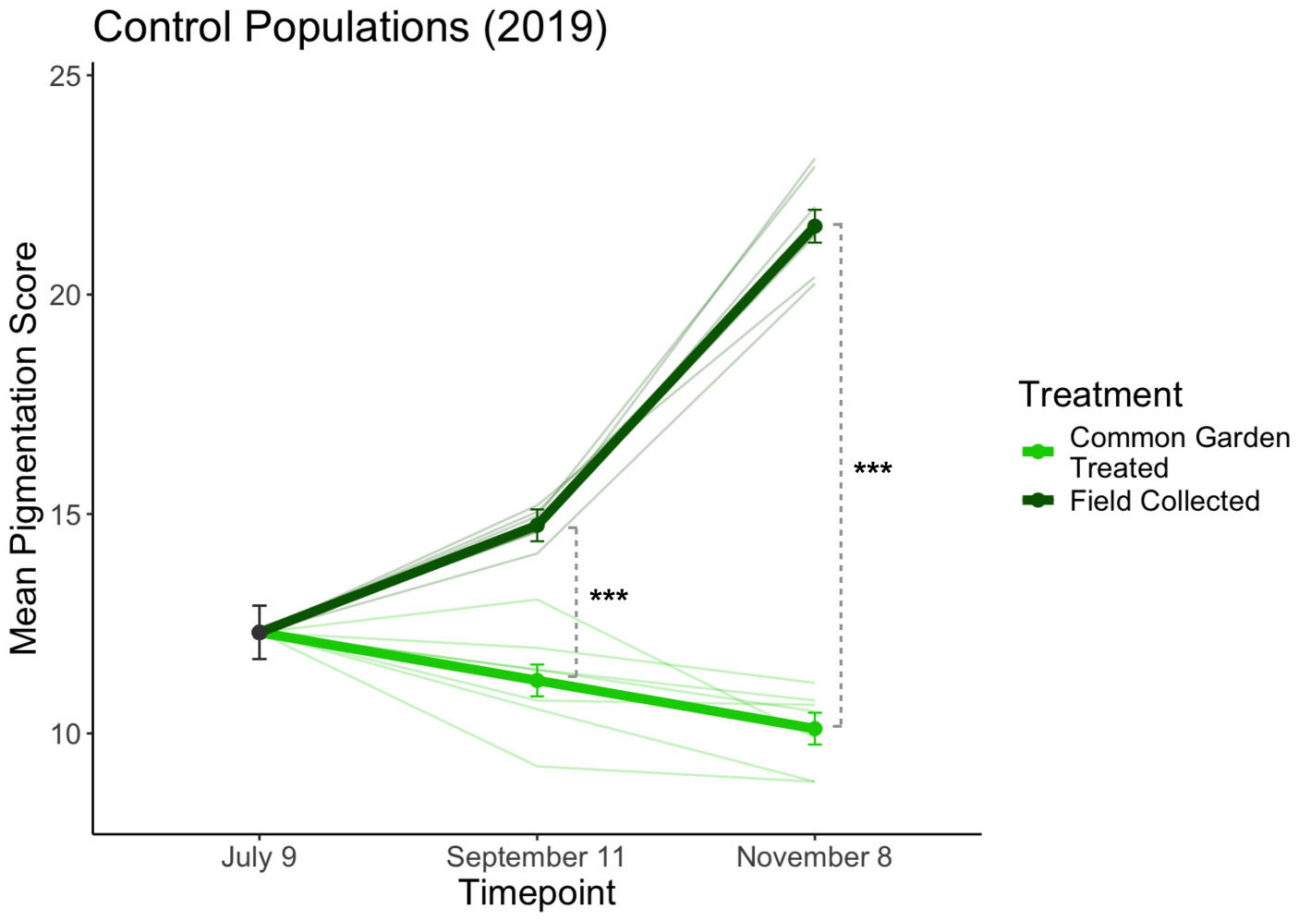
Pigmentation scores of control population females collected directly from field cages. During the 2019 experiment (interspecific competition), adult females were aspirated directly from each control cage and immediately preserved for pigmentation scoring in order to examine effects of plasticity on phenotype in the field. Here, mean pigmentation scores are plotted for females scored following two generations of laboratory, common garden treatment (25°C, 12L:12D), versus females scored following field collection (with no common garden treatment). Significant differences in mean pigmentation score between treatments are denoted (***p < .001). Mean pigmentation scores across each treatment (± SE) are plotted alongside individual cage scores (thin lines).

Importantly, *D. melanogaster* pigmentation is thermally plastic (David et al., 1990; Gibert et al., 2007; Lafuente et al., 2024), which raises the possibility that phenotypic plasticity may produce pigmentation patterns in outdoor populations that match seasonal temperature shifts. We had preserved flies directly from outdoor control cages in a prior experiment (2019), which enabled us to test whether plasticity modulates pigmentation patterns in the field. We compared melanization between the outdoor populations and flies from the same populations that underwent laboratory, common garden treatment (*Materials and methods* section). We found that field-caught individuals were significantly darker at both timepoints relative to common garden reared populations, and this difference was especially stark in the fall (Figure 2; Table S3B). Therefore, while we observed directional selection on light-associated alleles from summer to fall here (Table S3B) and in previous work (Berardi et al., 2025), phenotypic plasticity appears to enable field populations to exhibit darker pigmentation in response to cold conditions, consistent with the thermal melanism hypothesis.

### Intraspecific competition influences the adaptive trajectory of pigmentation

**Figure 3.**
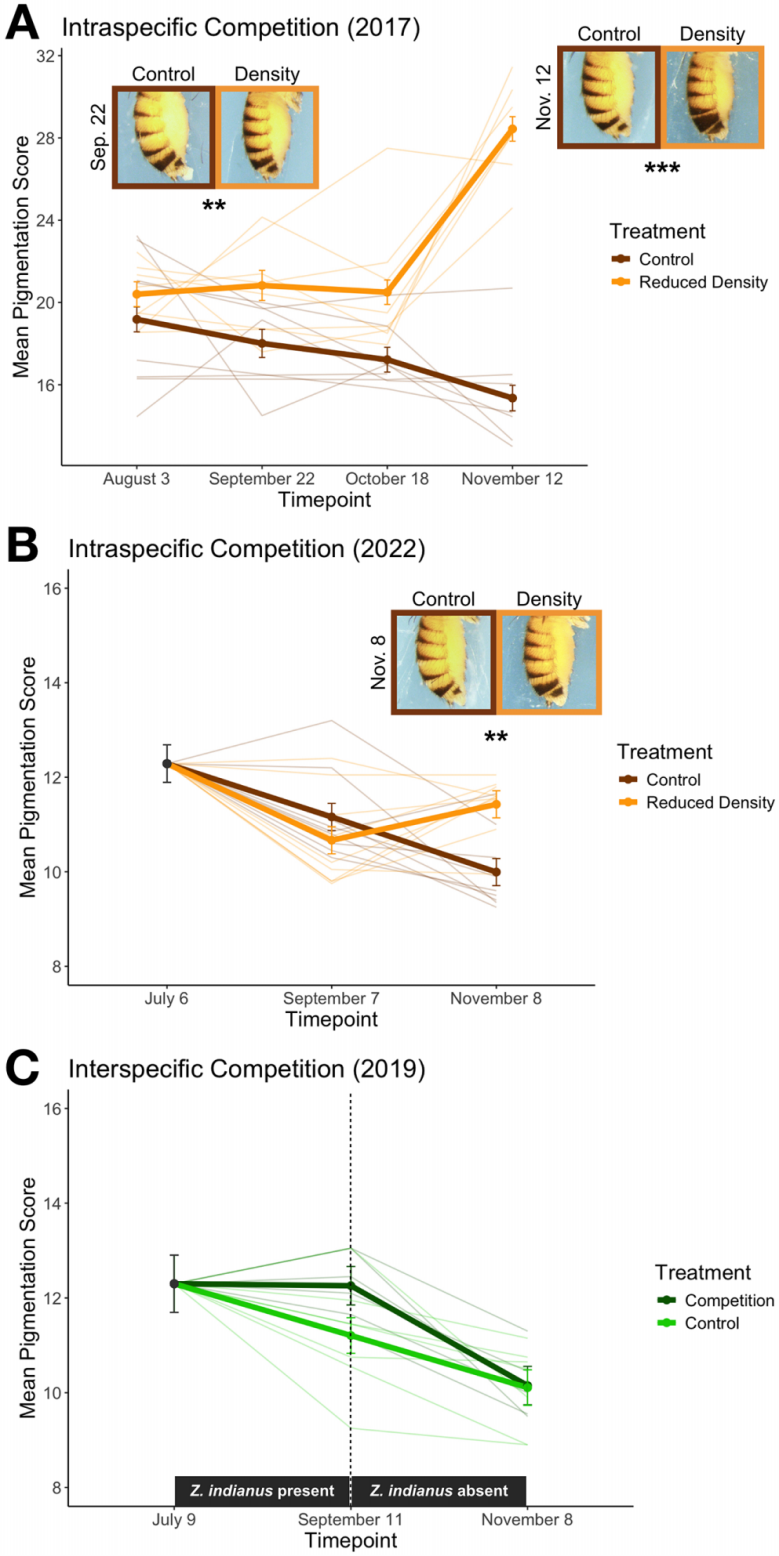
Manipulations of intraspecific competition drive differential pigmentation patterns across seasons. (A, B) Reducing *D. melanogaster* population density in two separate years of experimentation drove darker pigmentation relative to the control following the fall phase. Notably, all seven abdominal tergites were scored for the reduced density treatment in 2017 (panel B; see *Materials and methods* section), yielding a different *y*-axis scale. (C) Introducing *Z. indianus* as an interspecific competitor during the summer phase was associated with a nonsignificant increase in melanization. *Z. indianus* individuals were then removed from each competition treatment cage during the fall phase. In all plots, mean pigmentation scores across each treatment (± SE) are plotted alongside individual cage scores (thin lines). Representative images depicting the population mean are shown at the end of the summer and/or fall phase if differences in melanization between treatments are significant (***p < .001, **p < .01).

We next aimed to uncover whether other key components of the seasonal environment influence pigmentation patterns. Fluctuations in intraspecific competition associated with seasonal boom-bust dynamics (Bitter et al., 2026; Gleason et al., 2019), as well as seasonal shifts in the extent of interspecific competition (Grainger et al., 2021), have been associated with patterns of rapid evolution in *D. melanogaster*. Therefore, we tested whether modulating levels of intra- and interspecific competition drive differential pigmentation patterns across seasons. To manipulate the magnitude of intraspecific competition, we reduced *D. melanogaster* population density in two years (2017 and 2022) and compared evolutionary trajectories of pigmentation to control cages with unmanipulated density (*Materials and methods* section). In both years, lessening intraspecific competition yielded the same effect on pigmentation over time: reduced density populations evolved to be significantly darker in the fall relative to controls (light lines in Figure 3A, B). We measured pigmentation patterns at four timepoints in 2017, and we found that melanization varied significantly by treatment (Figure 3A; Table S4A; *F* = 49.38, *p* < .001), and effects of treatment varied with time (*F =* 94.30, *p* < .001). Contrasts showed a significant difference between control (*N* = 8 cages) and density treatments (*N* = 8 cages) at the end of the summer (*t* = −2.82, *p* = .007) and fall (*t =* −15.20, *p* < .001; Table S4B). In 2022, we similarly found that reducing density (*N* = 9 cages) altered the evolutionary trajectory of pigmentation relative to control (*N* = 9 cages), (Figure 3B; Table S5A; *F* = 17.02, *p* < .001), and reduced density populations were significantly darker than controls after the fall phase (Table S5B; *t =* −3.53, *p* = .004).

In addition to manipulating intraspecific competition, we tested for effects of interspecific competition by introducing a competitor fly, *Z. indianus*, to a subset of field mesocosms in 2019 (Grainger et al., 2021). However, our control (*N* = 7 cages) and interspecific competition (*N* = 7 cages) populations did not exhibit significantly different pigmentation patterns (Figure 3C; Table S6A). *Zaprionus indianus* were present in competition treatment mesocosms during the summer phase but absent during the fall phase, and we observed that both control and treatment populations evolved to be significantly lighter in the fall relative to summer but did not differ in pigmentation (Table S6B).

### Manipulations of diet and the gut microbiome yield differential shifts in melanization

**Figure 4.**
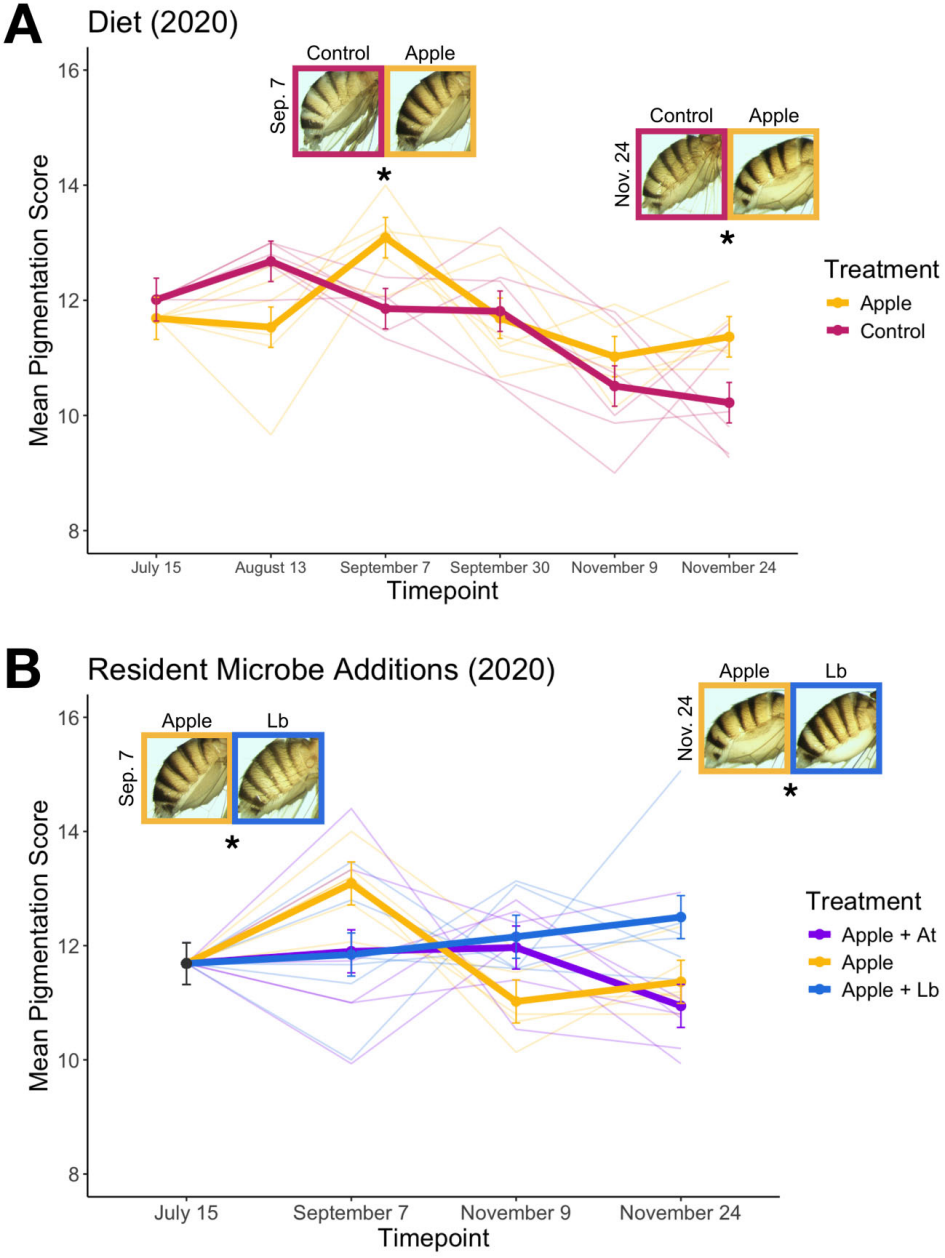
Distinct pigmentation patterns arise following manipulations of diet and the gut microbiome. (A) Populations subjected to our apple diet treatment exhibited shifts in melanization over time that differed significantly to shifts in the control populations. (B) Both resident microbe additions (*A. thailandicus* and *L. brevis*) produced melanization patterns that varied across seasons relative to control populations. Here, the control populations are the apple-based food treatment (shown in A), as apple food was supplemented with each microbe in this experiment. The *A. thailandicus* treatment is denoted as “Apple + At,” and the *L. brevis* treatment is denoted as “Apple + Lb.” In all plots, mean pigmentation scores across each treatment (± SE) are shown alongside individual cage scores (thin lines). Representative images depicting the population mean for each treatment are shown at the end of the summer and fall (*p < .05).

Previous work has also demonstrated that diet (Beltz et al., 2025) and the gut microbiome (Rudman et al., 2019) represent key drivers of seasonal adaptation in *D. melanogaster*, and we examined whether these factors influence the evolution of pigmentation during our 2020 experiment. We first found that feeding flies a lower quality, apple-based diet (Beltz et al., 2025) altered the seasonal trajectory of pigmentation adaptation relative to the higher quality control diet (Figure 4A; Table S7A; *F* = 4.14, *p* = .003). Populations evolving on the apple diet (*N* = 6 cages) exhibited darker pigmentation relative to the control diet (*N* = 6 cages) at the end of summer (*t* = −2.49, *p* = .028) and fall (*t* = −2.31, *p* = .028; Table S7B), but patterns of melanization fluctuated between treatments at intermediate timepoints (Figure 4A). We then altered the gut microbiome of *D. melanogaster* populations by supplementing the apple diet with one of two colonizing microbes (Figure 4B). We found that treating populations with either *A. thailandicus* (*N* = 6 cages; Table S8A; *F* = 4.73, *p* = .009) or *L. brevis* (*N* = 6 cages; Table S9A; *F* = 8.37, *p* < .001) altered the trajectory of melanization over time relative to controls. Interestingly, effects on pigmentation patterns at specific timepoints differed for each microbe (Figure 4B; Table S8B; Table S9B).

## Discussion

We utilized a field-based, experimental evolution approach to examine how multiple environmental agents of selection influence adaptive dynamics of pigmentation over ecological timescales (Hairston et al., 2005). We first observed that our unmanipulated control populations consistently evolved to be less melanized over time, showing a parallel response suggesting directional selection on light-associated alleles across all four years of experimentation (Tables S2B–S8B) despite inter-year variation in the composition of our outbred founding population (*Materials and methods* section). This finding was concordant with our expectations based on previous work, which demonstrated that temperate *D. melanogaster* populations repeatedly evolve lighter pigmentation following the summer growing season (Berardi et al., 2025). We then explored the complexity of the seasonal selective landscape for pigmentation to identify environmental agents that drive parallel evolutionary patterns indicative of adaptation. Under the simplest scenario, we anticipated that patterns of melanization would respond to manipulations of temperature, in line with the thermal melanism hypothesis (Clusella-Trullas et al., 2007). However, while we confirmed increasing the temperature drove evolution of lighter pigmentation during the summer, as would be predicted if lessening melanization minimizes overheating, we did not observe a genetic shift to darker pigmentation to promote heating during the fall, when mean daily temperatures in Philadelphia typically decrease to 9°C–15°C (Table S10; Bitter et al., 2024; Palecki et al., 2021; Rudman et al., 2022). Instead, plasticity appears to drive darker pigmentation in response to colder fall temperatures as predicted under thermal melanism, creating a mismatch between patterns of selection on alleles underlying variance in pigmentation and the actual expression of melanism phenotype in the field. Overall, we found that manipulating several key environmental parameters drove differential pigmentation patterns over seasons. Thus, our findings illustrate that the selective landscape of *D. melanogaster* pigmentation is multifaceted, and temperature gradients alone may not be sufficient to explain melanization patterns in wild populations.

Both the parallelism in evolutionary responses across replicates and our ability to control for confounding effects of migration and demography with our field mesocosm system enable us to conclude that observed changes in pigmentation are most likely driven by adaptive evolution. However, whether pigmentation is under direct or indirect selection under each set of conditions remains an open question. Our temperature manipulation provided some evidence that pigmentation may be under direct selection to produce a thermoregulatory benefit during the summer. We observed that warming treatment populations evolved to be lighter than control populations following the summer, concordant with our expectations if lighter pigmentation produces a cooler body temperature in the field (Freoa et al., 2023). This finding aligns with a breadth of previous work showing that lighter pigmentation covaries with warmer temperatures over latitudinal (Telonis-Scott et al., 2011), altitudinal (Bastide et al., 2014), and seasonal gradients (Berardi et al., 2025). However, we did not measure the body temperature of warming and control treatment flies, and we observed continued selection for light pigmentation as temperatures decreased during the fall, contrary to predictions of thermal melanism. Populations instead expressed dark pigmentation in the field via plasticity, indicating that allele frequency change was decoupled from realized phenotype. Unlike systems where variation in pigmentation can be experimentally created to directly test fitness consequences (e.g., Kingsolver, 1987), laboratory validation of thermal melanism remains challenging in *Drosophila*. We also cannot exclude the possibility that polyethylene wrap may have lessened UV radiation in our warming cages (*Materials and methods* section), potentially contributing to the reduced melanization observed during the summer (Bastide et al., 2014). Therefore, we cannot presently conclude whether *D. melanogaster* melanization adapted to directly support thermoregulation.

We considered whether the reduction in melanization observed in November could be attributed to summer-evolved flies persisting into the fall, as previous work suggests wild *D. melanogaster* individuals can live for > 2 months (Behrman et al., 2015). However, we believe this is likely not the case, as fecundity profiles in *D. melanogaster* show reduced egg output with age (Miller et al., 2014; Rogina et al., 2007), suggesting that adults from the most recent generation reaching their reproductive peak were the primary contributors to populations sampled for phenotyping (Bitter et al., 2024). Instead, it appears more likely that adaptive plasticity plays a role in modulating selection on darker pigmentation during the fall. Our finding that populations collected directly from field mesocosms were significantly darker than common garden populations at both timepoints (Figure 2) indicates that plasticity drives phenotypic responses to temperature shifts in the field. Thus, plasticity may provide an alternative mechanism for improving fitness while mitigating pleiotropic effects associated with selection on melanization. Notably, reaction norms of *Drosophila* pigmentation to rearing temperature have been shown to be highly non-linear, with a sigmoid shape (Gibert et al., 1998); this suggests that the effects of thermal plasticity along an environmental gradient are not constant but are instead dependent on how a thermal shift aligns with the trait’s reaction norm. Therefore, the degree to which plasticity affects dynamics of selection and phenotypic expression may vary with seasonal temperature profiles. Recent work also found that pigmentation plasticity in *Drosophila* scales positively with increased heterozygosity for variants associated with light and dark pigmentation, and that plastic patterns are associated with a reversal of dominance between light and dark alleles based on rearing temperature (David et al., 2026). This suggests phenotypic plasticity may provide a mechanism for maintaining standing variation for pigmentation-related loci in seasonal fly populations. We do know that *D. melanogaster* populations consistently evolve darker pigmentation by spring from previous work (Berardi et al., 2025), and the dynamics driving the genetic reversion to darker pigmentation during the overwintering phase remain unresolved.

Notably, we found that pigmentation patterns evolved in response to components of the seasonal environment that were previously not connected to melanization in *D. melanogaster*: intraspecific competition, diet, and the gut microbiome. This suggests that evolution of pigmentation is either a component of adaptation to a wide range of stressors or is indirectly driven by correlations with other traits under seasonally varying selection. The loci that regulate insect pigmentation are notoriously pleiotropic (Wittkopp & Beldade, 2009), and *D. melanogaster* pigmentation genes have been implicated in affecting mating behavior (Drapeau et al., 2003; Wilson et al., 1976), morphology (Godt et al., 1993; Kopp et al., 2000), cuticle hardening and immunity (Hodgetts & O’Keefe, 2006; Jacobs, 1985), circadian activity (Shaw et al., 2000), vision (True et al., 2005), and cuticular hydrocarbon composition (Massey et al., 2019). Numerous fitness-related phenotypes evolve seasonally in *D. melanogaster* (Behrman et al., 2015; Erickson et al., 2020; Rudman et al., 2022; Schmidt & Conde, 2006), and these patterns appear influenced by life history trade-offs between reproduction and survival (Bitter et al., 2026; Flatt et al., 2013). Several traits with pleiotropic associations to pigmentation are known to cycle seasonally, including innate immunity (Behrman et al., 2018), cuticular hydrocarbon content (Rajpurohit et al., 2017), and desiccation tolerance (Rajpurohit et al., 2018). Therefore, pigmentation may be under frequent indirect selection due to genetic correlations, providing an explanation for its responsiveness to a breadth of ecological manipulations. Finally, patterns of linkage disequilibrium associated with chromosomal inversions in *D. melanogaster* (Kapun et al., 2016) may also influence the seasonal evolution of pigmentation.

Interestingly, both intraspecific competition manipulations yielded pigmentation shifts as predicted by thermal melanism: reducing population density drove darker pigmentation in the fall. There is evidence that melanin is metabolically costly to produce across both insects (Ethier et al., 2015; Roff & Fairbairn, 2013; Stoehr, 2006) and vertebrates (Britton & Davidowitz, 2023; Stoehr, 2006), which provides a possible explanation for this pattern. If melanin synthesis is costly under stressful conditions, alleviating intraspecific competition for non-substitutable resources may yield a higher capacity to produce melanin, as observed. Conversely, melanization may be constrained by increased competition in control populations, imposing a metabolic trade-off that limits pigment synthesis. Therefore, it is possible that lessening competition reduced fitness costs of melanin production, leading flies to evolve darker pigmentation and improve thermoregulation in the fall. Importantly, however, results of our diet manipulation were not aligned with the hypothesis that melanin is costly in resource limited environments. The apple diet represented a reduction in nutritional quality relative to the control diet (Beltz et al., 2025); thus, if melanin production is costly, we would have anticipated flies reared on apple food to consistently be lighter than flies reared on control food across all timepoints, which was not the case. We also failed to see significant change in pigmentation in our interspecific competition treatment relative to the control, despite the resource environment varying between treatments.

Altogether, we demonstrate that adaptive dynamics of *D. melanogaster* pigmentation are nuanced, which provides exciting opportunities for future exploration. It is clear temperature is not the sole driver of pigmentation in *D. melanogaster*, so revisiting melanization patterns in the context of life history theory and energetic trade-offs represents important steps for yielding insight into determinants of phenotype. Pigmentation patterns were responsive to a range of environmental manipulations, and defining whether these shifts are largely driven by direct fitness benefits or indirect selection remains a key area of investigation. Our finding that flies evolve to be lighter across seasons, yet exhibit darker pigmentation in the field via plasticity, suggests that plasticity modulates the adaptive dynamics of this trait. Finally, examining agents of selection acting on other complex traits will enable us to understand whether the selective landscape for pigmentation is uniquely multifaceted, or if it represents the standard for how patterns of complex trait adaptation are shaped in dynamic environments.

## Supplementary Material

qpag038_Supplemental_File

## Data Availability

Raw data and scripts associated with this project have been uploaded to the following GitHub repository: https://github.com/skylerberardi/Papers/tree/main/Berardi_2026_Evolution
