# Supplementary material for: Seasonal evolution of Drosophila melanogaster abdominal pigmentation is associated with a multifarious selective landscape
Source: Evolution. 2026 Mar 11;80(5):1111–21. doi: 10.1093/evolut/qpag038 (PMC13168820; doi:10.1093/evolut/qpag038)
Supplement: qpag038_Supplemental_File [file qpag038_supplemental_file.pdf]

Supplementary Materials for

Seasonal evolution of *Drosophila melanogaster* abdominal pigmentation is associated with a multifarious selective landscape

Authors: Skyler Berardi*, Jack K. Beltz, Seth M. Rudman, Tess N. Grainger, Jonathan M. Levine, Hayes Oken, and Paul Schmidt*

* **Corresponding Author Information:** S. B. (berardis@sas.upenn.edu) and P. S. (schmidtp@sas.upenn.edu); 3740 Hamilton Walk, Philadelphia, PA 19104, USA

This file includes:

Tables S1 to S10

References

Table S1. Mean daily high temperatures across control and warming cages. Temperature measurements were taken with data loggers (HOBO Pendant MX2202) throughout the 2022 experiment at 10-minute intervals, and the daily high temperature for each cage was calculated by averaging temperatures recorded from 2:00 PM – 4:00 PM. Two outlier control cages ("E2" and "E3") were removed from the analysis; the peach trees in these cages were replaced at the beginning of the season and provided less shade, and the cages exhibited elevated temperatures relative to the other control cages. The mean daily high temperature was calculated at each date across the control cages and the warming cages, and we then determined the temperature difference at each date between the warming and control treatments. We found that across the entire season, mean daily high temperatures in the warming treatment were 0.86 +/- 0.03°C greater than the control. This difference was 0.99 +/- 0.03°C across the summer phase, and 0.73 +/- 0.04°C across the fall phase (mean +/- standard error).

Date	Phase	Mean Daily High Temperature °C (Control Cages)	Standard Error °C (Control Cages)	Mean Daily High Temperature °C (Warming Cages)	Standard Error °C (Warming Cages)	Temperature Difference °C (Warming - Control)
2022-07-06	Summer	32.8671	0.2266	33.7026	0.1933	0.8355
2022-07-07	Summer	29.9402	0.4556	30.8657	0.3170	0.9255
2022-07-08	Summer	32.4856	0.3350	33.4321	0.2223	0.9465
2022-07-09	Summer	25.1634	0.2381	25.7494	0.2060	0.5860
2022-07-10	Summer	29.0439	0.2366	29.7030	0.1839	0.6591
2022-07-11	Summer	34.1582	0.7677	35.4862	0.3918	1.3280
2022-07-12	Summer	36.8027	0.5755	37.7758	0.3122	0.9731
2022-07-13	Summer	37.3931	0.5308	38.7172	0.2784	1.3241
2022-07-14	Summer	36.7996	0.4782	37.9645	0.2667	1.1648
2022-07-15	Summer	35.5024	0.4475	36.5917	0.2757	1.0893
2022-07-16	Summer	34.3160	0.2634	35.2828	0.1588	0.9668
2022-07-17	Summer	32.9141	0.3106	33.8838	0.1996	0.9697
2022-07-18	Summer	35.5540	0.2732	36.2481	0.1659	0.6941
2022-07-19	Summer	37.4396	0.4863	38.6517	0.2621	1.2121
2022-07-20	Summer	40.0299	0.4434	41.2265	0.2764	1.1966
2022-07-21	Summer	38.7899	0.3441	39.7794	0.2218	0.9895
2022-07-22	Summer	40.0677	0.4415	41.3079	0.2875	1.2402
2022-07-23	Summer	40.2761	0.4198	41.5482	0.2708	1.2721
2022-07-24	Summer	40.1316	0.3640	41.2853	0.2138	1.1537
2022-07-25	Summer	33.5075	0.1552	33.9596	0.1545	0.4521
2022-07-26	Summer	27.9989	0.1743	28.6420	0.1243	0.6431
2022-07-27	Summer	34.1112	0.2214	34.8733	0.2181	0.7621
2022-07-28	Summer	35.8109	0.1957	36.5611	0.1686	0.7502
2022-07-29	Summer	31.7231	0.2363	32.4967	0.1359	0.7736
2022-07-30	Summer	34.8727	0.4449	36.2087	0.3356	1.3360
2022-07-31	Summer	35.1229	0.2848	36.1587	0.1814	1.0357
2022-08-01	Summer	26.1180	0.2326	26.8696	0.1643	0.7516
2022-08-02	Summer	35.4238	0.3584	36.6572	0.2722	1.2334
2022-08-03	Summer	36.4825	0.4143	37.7146	0.3184	1.2321
2022-08-04	Summer	39.1949	0.3620	40.3967	0.2605	1.2018
2022-08-05	Summer	34.3987	0.3122	35.5934	0.2251	1.1947

2022-08-06	Summer	34.9922	0.3369	35.9132	0.2062	0.9210
2022-08-07	Summer	37.9033	0.4079	39.0215	0.2692	1.1182
2022-08-08	Summer	37.5213	0.2807	38.3339	0.2377	0.8126
2022-08-09	Summer	38.7210	0.2936	39.8596	0.2709	1.1387
2022-08-10	Summer	33.9314	0.2280	34.8661	0.1685	0.9347
2022-08-11	Summer	33.6005	0.3472	34.7450	0.2730	1.1445
2022-08-12	Summer	31.7880	0.3147	32.8383	0.2396	1.0503
2022-08-13	Summer	31.4693	0.4353	32.7858	0.3408	1.3166
2022-08-14	Summer	32.5096	0.3713	33.7507	0.2518	1.2411
2022-08-15	Summer	30.2650	0.3236	31.1687	0.1971	0.9037
2022-08-16	Summer	32.4069	0.4372	33.5958	0.3590	1.1888
2022-08-17	Summer	28.8319	0.2133	29.4003	0.0954	0.5684
2022-08-18	Summer	33.5842	0.3377	34.8053	0.2818	1.2212
2022-08-19	Summer	36.6944	0.3166	38.0161	0.2892	1.3217
2022-08-20	Summer	36.5514	0.2959	37.8139	0.2863	1.2625
2022-08-21	Summer	31.9994	0.1588	32.6500	0.1207	0.6506
2022-08-22	Summer	31.3559	0.3146	32.3719	0.1724	1.0161
2022-08-23	Summer	31.0168	0.3072	32.1082	0.2136	1.0914
2022-08-24	Summer	34.1863	0.3198	35.3070	0.2887	1.1208
2022-08-25	Summer	34.9069	0.2362	35.8933	0.2470	0.9864
2022-08-26	Summer	35.2813	0.2381	36.1770	0.2524	0.8957
2022-08-27	Summer	34.3089	0.2339	35.1652	0.2500	0.8564
2022-08-28	Summer	31.3896	0.2031	31.9143	0.1220	0.5247
2022-08-29	Summer	36.0683	0.2825	37.2332	0.2700	1.1649
2022-08-30	Summer	35.6015	0.2088	36.4757	0.1965	0.8742
2022-08-31	Summer	30.1138	0.2311	30.9797	0.1961	0.8658
2022-09-01	Summer	33.1853	0.3459	34.3322	0.3407	1.1470
2022-09-02	Summer	30.9044	0.4032	32.0363	0.3737	1.1319
2022-09-03	Summer	32.3196	0.2380	33.2184	0.2305	0.8988
2022-09-04	Summer	33.4784	0.2013	34.2588	0.1947	0.7803
2022-09-05	Summer	33.6357	0.2175	34.3855	0.1879	0.7497
2022-09-06	Summer	22.0986	0.1544	22.5215	0.0774	0.4229
2022-09-07	Fall	22.0552	0.0732	22.3642	0.0661	0.3090
2022-09-08	Fall	27.4668	0.2029	28.0708	0.2372	0.6040
2022-09-09	Fall	29.4044	0.3340	30.1928	0.3288	0.7883
2022-09-10	Fall	29.1322	0.1898	29.7987	0.2513	0.6665
2022-09-11	Fall	22.9454	0.0729	23.3521	0.0400	0.4068
2022-09-12	Fall	31.0066	0.3411	32.0033	0.2506	0.9968
2022-09-13	Fall	30.0433	0.3156	31.0619	0.2599	1.0186
2022-09-14	Fall	28.5265	0.3648	29.4257	0.3720	0.8992
2022-09-15	Fall	26.2952	0.4163	27.1381	0.4010	0.8429
2022-09-16	Fall	27.2344	0.3402	28.1462	0.3663	0.9119
2022-09-17	Fall	29.9060	0.2283	30.7505	0.2628	0.8445
2022-09-18	Fall	30.8145	0.2211	31.6728	0.3073	0.8583
2022-09-19	Fall	31.6215	0.1571	32.2202	0.2265	0.5987
2022-09-20	Fall	29.2548	0.3510	30.0490	0.3649	0.7942
2022-09-21	Fall	30.3758	0.2994	31.2696	0.3121	0.8938
2022-09-22	Fall	18.5991	0.1411	19.1304	0.0970	0.5313

2022-09-23	Fall	20.3795	0.3299	21.2405	0.3659	0.8610
2022-09-24	Fall	22.9295	0.3684	23.9221	0.4063	0.9926
2022-09-25	Fall	23.2155	0.1720	23.7156	0.1859	0.5000
2022-09-26	Fall	25.1624	0.2225	25.8693	0.2283	0.7070
2022-09-27	Fall	23.4254	0.2662	24.2288	0.3013	0.8034
2022-09-28	Fall	21.2181	0.1729	21.6826	0.1670	0.4645
2022-09-29	Fall	21.7110	0.3261	22.4846	0.3046	0.7736
2022-09-30	Fall	18.0767	0.1047	18.2740	0.0491	0.1973
2022-10-01	Fall	15.6390	0.1102	15.9335	0.0357	0.2945
2022-10-02	Fall	12.5374	0.0325	12.7021	0.0193	0.1647
2022-10-03	Fall	10.2765	0.1084	10.7998	0.0847	0.5233
2022-10-04	Fall	10.5497	0.0512	10.7839	0.0424	0.2342
2022-10-05	Fall	16.5098	0.1558	16.8912	0.0750	0.3815
2022-10-06	Fall	24.0996	0.4048	25.3093	0.4555	1.2097
2022-10-07	Fall	25.5669	0.3607	26.6114	0.4100	1.0445
2022-10-08	Fall	16.5333	0.3601	17.5502	0.4503	1.0170
2022-10-09	Fall	18.0797	0.3464	19.1534	0.4529	1.0737
2022-10-10	Fall	20.8223	0.3515	21.9213	0.4544	1.0990
2022-10-11	Fall	23.7692	0.3200	24.7766	0.4119	1.0074
2022-10-12	Fall	22.4571	0.1891	23.1285	0.2367	0.6714
2022-10-13	Fall	20.7442	0.0521	21.0029	0.0373	0.2588
2022-10-14	Fall	20.3616	0.4288	21.6969	0.5259	1.3353
2022-10-15	Fall	22.8504	0.2662	23.8737	0.4180	1.0233
2022-10-16	Fall	19.4117	0.4266	20.6252	0.4935	1.2135
2022-10-17	Fall	21.4980	0.1708	22.2733	0.3036	0.7753
2022-10-18	Fall	14.4802	0.2807	15.6217	0.4374	1.1415
2022-10-19	Fall	12.9194	0.0949	13.2173	0.1286	0.2979
2022-10-20	Fall	16.3974	0.2627	17.3635	0.5046	0.9661
2022-10-21	Fall	18.4541	0.3307	19.5765	0.5679	1.1224
2022-10-22	Fall	20.1752	0.3308	21.3159	0.5774	1.1407
2022-10-23	Fall	17.4960	0.0603	17.6932	0.0549	0.1973
2022-10-24	Fall	16.1651	0.0877	16.3979	0.0434	0.2328
2022-10-25	Fall	21.8243	0.1784	22.3297	0.1515	0.5053
2022-10-26	Fall	20.3182	0.0662	20.5823	0.0689	0.2640
2022-10-27	Fall	18.1272	0.2647	19.0792	0.5119	0.9519
2022-10-28	Fall	15.2825	0.1195	15.6515	0.1310	0.3690
2022-10-29	Fall	15.9460	0.2919	17.0235	0.5328	1.0776
2022-10-30	Fall	17.8075	0.2550	18.8869	0.4937	1.0794
2022-10-31	Fall	18.4347	0.0636	18.6561	0.0643	0.2214
2022-11-01	Fall	21.3621	0.2237	22.2180	0.2987	0.8559
2022-11-02	Fall	20.7360	0.2157	21.6695	0.3923	0.9335
2022-11-03	Fall	20.8683	0.2478	21.8836	0.4639	1.0153
2022-11-04	Fall	21.8315	0.2208	22.7952	0.4279	0.9637
2022-11-05	Fall	25.2885	0.1013	25.7796	0.1768	0.4910
2022-11-06	Fall	23.9980	0.0865	24.2316	0.0703	0.2336
2022-11-07	Fall	24.0061	0.2928	24.5081	0.2400	0.5019
2022-11-08	Fall	15.1232	0.2386	15.6634	0.2310	0.5402

Table S2. Statistical analyses of the warming treatment. (A) We ran a linear mixed effects model to determine how timepoint, treatment (control vs. warmed temperature), and their interaction influenced pigmentation patterns. Cage was included in the model as a random effect. (B) Planned comparisons showing whether the control and warming treatments exhibited significant shifts in pigmentation from summer to fall, and whether there were significant differences in pigmentation between treatments at the end of the summer (Sep. 7) and fall (Nov. 8) phases. Raw *p*-values and 95% confidence intervals are reported, as well as *p*-values following Holm correction for multiple comparisons.

A **Increased Temperature (2022)**

Pigmentation Score ~ Timepoint * Treatment + (1 | Cage)

	Sum Sq	Mean Sq	Num DF	Den DF	F value	*p*-value
**Timepoint**	43.022	43.022	1	700	3.8108	0.0513
**Treatment**	37.469	37.469	1	16	3.3189	0.0872
**Timepoint x Treatment**	82.689	82.689	1	700	7.3244	**0.0070**

B Contrasts

Contrast	Estimate	SE	Df	Lower CL	Upper CL	*t* ratio	*p*-value	Adjusted *p*-value (Holm)
**Control: Sep. 7 - Nov. 8**	1.167	0.354	700	0.471	1.862	3.294	**0.0010**	**0.0041**
**Warming: Sep. 7 - Nov. 8**	-0.189	0.354	700	-0.884	0.506	-0.533	0.5940	1.0000
**Sep. 7: Control - Warming**	1.144	0.358	60	0.428	1.861	3.195	**0.0022**	**0.0067**
**Nov. 8: Control - Warming**	-0.211	0.358	60	-0.928	0.505	-0.589	0.5579	1.0000

Table S3. Analyzing the effects of plasticity on pigmentation in control populations collected directly from field cages, versus control populations subjected to laboratory, common garden treatment. (A) We ran a linear mixed effects model to determine how timepoint, treatment (field collected vs. common garden treated), and their interaction influenced pigmentation patterns in the 2019 control cages. Cage was included in the model as a random effect. (B) Planned comparisons showing change in pigmentation from summer to fall in the common garden treated and field collected (outdoor) populations, and whether there were significant differences in pigmentation between the common garden treated (CG) and field collected populations due to plasticity at the end of the summer (Sep. 11) and fall (Nov. 8) phases. Raw *p*-values and 95% confidence intervals are reported, as well as *p*-values following Holm correction for multiple comparisons.

A Field Collected vs. Common Garden Treated: Control Cages (2019)

Pigmentation Score ~ Timepoint * Treatment + (1 | Cage)

	Sum Sq	Mean Sq	Num DF	Den DF	F value	*p*-value
**Timepoint**	1118.4	1118.4	1	535.02	85.306	**< .0001**
**Treatment**	4278.0	4278.0	1	12.12	326.310	**< .0001**
**Timepoint x Treatment**	2144.8	2144.8	1	535.02	163.599	**< .0001**

B Contrasts

Contrast	Estimate	SE	Df	Lower CL	Upper CL	*t* ratio	*p*-value	Adjusted *p*-value (Holm)
**Common Garden: Sep. 7 - Nov. 24**	1.10	0.433	533.0	0.25	1.95	2.542	**0.0113**	**0.0113**
**Field Collected: Sep. 7 - Nov. 24**	-6.82	0.443	536.6	-7.69	-5.95	-15.396	**<.0001**	**<.0001**
**Sep. 7: CG - Field**	-3.54	0.513	28.0	-4.59	-2.48	-6.886	**<.0001**	**<.0001**
**Nov. 24: CG - Field**	-11.45	0.522	29.6	-12.52	-10.39	-21.944	**<.0001**	**<.0001**

Table S4. Statistical analyses of the reduced intraspecific competition treatment (2017). (A) We ran a linear mixed effects model to determine how timepoint, treatment (control vs. reduced population density), and their interaction influenced pigmentation patterns. Cage was included in the model as a random effect. (B) Planned comparisons showing whether the control and reduced density treatments exhibited significant shifts in pigmentation from summer to fall, and whether there were significant differences in pigmentation between treatments at the end of the summer (Sep. 22) and fall (Nov. 12) phases. Raw *p*-values and 95% confidence intervals are reported, as well as *p*-values following Holm correction for multiple comparisons.

A Intraspecific Competition (2017)

Pigmentation Score ~ Timepoint * Treatment + (1 | Cage)

	Sum Sq	Mean Sq	Num DF	Den DF	F value	*p*-value
**Timepoint**	1532.6	510.87	3	1079.96	23.045	**< .0001**
**Treatment**	1094.6	1094.57	1	13.88	49.375	**< .0001**
**Timepoint x Treatment**	6271.3	2090.42	3	1079.96	94.296	**< .0001**

B Contrasts

Contrast	Estimate	SE	Df	Lower CL	Upper CL	*t* ratio	*p*-value	Adjusted *p*-value (Holm)
**Control: Sep. 22 - Nov. 12**	2.66	0.631	1082.9	1.42	3.896	4.209	**<.0001**	**0.0001**
**Reduced Density: Sep. 22 - Nov. 12**	-7.61	0.676	1081.5	-8.93	-6.281	-11.256	**<.0001**	**<.0001**
**Sep. 22: Control - Density**	-2.82	1.001	48.0	-4.83	-0.805	-2.815	**0.0071**	**0.0071**
**Nov. 12: Control - Density**	-13.08	0.861	27.8	-14.85	-11.319	-15.202	**<.0001**	**<.0001**

Table S5. Statistical analyses of the reduced intraspecific competition treatment (2022). (A) We ran a linear mixed effects model to determine how timepoint, treatment (control vs. reduced population density), and their interaction influenced pigmentation patterns. Cage was included in the model as a random effect. (B) Planned comparisons showing whether the control and reduced density treatments exhibited significant shifts in pigmentation from summer to fall, and whether there were significant differences in pigmentation between treatments at the end of the summer (Sep. 7) and fall (Nov. 8) phases. Raw *p*-values and 95% confidence intervals are reported, as well as *p*-values following Holm correction for multiple comparisons.

A **Intraspecific Competition (2022)**

Pigmentation Score ~ Timepoint * Treatment + (1 | Cage)

	Sum Sq	Mean Sq	Num DF	Den DF	F value	*p*-value
**Timepoint**	7.401	7.401	1	700	0.7532	0.3858
**Treatment**	19.700	19.700	1	16	2.0047	0.1760
**Timepoint x Treatment**	167.235	167.235	1	700	17.0182	**< .0001**

B Contrasts

Contrast	Estimate	SE	Df	Lower CL	Upper CL	*t* ratio	*p*-value	Adjusted *p*-value (Holm)
**Control: Sep. 7 - Nov. 8**	1.167	0.330	700.0	0.518	1.815	3.531	**0.0004**	**0.0018**
**Reduced Density: Sep. 7 - Nov. 8**	-0.761	0.330	700.0	-1.410	-0.112	-2.303	**0.0216**	**0.0431**
**Sep. 7: Control - Density**	0.494	0.406	35.6	-0.328	1.317	1.219	0.2309	0.2309
**Nov. 8: Control - Density**	-1.433	0.406	35.6	-2.256	-0.610	-3.534	**0.0012**	**0.0035**

Table S6. Statistical analyses of the interspecific competition treatment. (A) We ran a linear mixed effects model to determine how timepoint, treatment (control vs. competition with *Z. indianus*), and their interaction influenced pigmentation patterns. Cage was included in the model as a random effect. (B) Planned comparisons showing whether the control and competition treatments exhibited significant shifts in pigmentation from summer to fall, and whether there were significant differences in pigmentation between treatments at the end of the summer (Sep. 11) and fall (Nov. 8) phases. Raw *p*-values and 95% confidence intervals are reported, as well as *p*-values following Holm correction for multiple comparisons.

A Interspecific Competition (2019)

Pigmentation Score ~ Timepoint * Treatment + (1 | Cage)

	Sum Sq	Mean Sq	Num DF	Den DF	F value	*p*-value
**Timepoint**	332.56	332.56	1	505	23.0331	**< .0001**
**Treatment**	22.30	22.30	1	11	1.5445	0.2398
**Timepoint x Treatment**	32.85	32.85	1	505	2.2751	0.1321

B Contrasts

Contrast	Estimate	SE	Df	Lower CL	Upper CL	*t* ratio	*p*-value	Adjusted *p*-value (Holm)
**Control: Sep. 11 - Nov. 8**	1.1000	0.454	505.0	0.208	1.9923	2.422	**0.0158**	**0.0474**
**Competition: Sep. 11 - Nov. 8**	2.1083	0.491	505.0	1.145	3.0721	4.298	**<.0001**	**0.0001**
**Sep. 11: Control - Competition**	-1.0512	0.553	27.1	-2.185	0.0825	-1.902	0.0678	0.1357
**Nov. 8: Control - Competition**	-0.0429	0.553	27.1	-1.177	1.0909	-0.078	0.9388	0.9388

Table S7. Statistical analyses of the diet treatment. (A) We ran a linear mixed effects model to determine how timepoint, treatment (control vs. apple food), and their interaction influenced pigmentation patterns. Cage was included in the model as a random effect. (B) Planned comparisons showing whether the control and apple treatments exhibited significant shifts in pigmentation from summer to fall, and whether there were significant differences in pigmentation between treatments at the end of the summer (Sep. 7) and fall (Nov. 24) phases. Raw *p*-values and 95% confidence intervals are reported, as well as *p*-values following Holm correction for multiple comparisons.

A Diet (2020)

Pigmentation Score ~ Timepoint * Treatment + (1 | Cage)

	Sum Sq	Mean Sq	Num DF	Den DF	F value	*p*-value
**Timepoint**	428.36	107.089	4	880	10.1293	**< .0001**
**Treatment**	19.12	19.123	1	10	1.8088	0.2084
**Timepoint x Treatment**	175.07	43.768	4	880	4.1399	**0.0025**

B Contrasts

Contrast	Estimate	SE	Df	Lower CL	Upper CL	*t* ratio	*p*-value	Adjusted *p*-value (Holm)
**Control: Sep. 7 - Nov. 24**	1.63	0.485	880	0.682	2.585	3.370	**0.0008**	**0.0024**
**Apple: Sep. 7 - Nov. 24**	1.72	0.485	880	0.771	2.674	3.553	**0.0004**	**0.0016**
**Sep. 7: Control - Apple**	-1.23	0.496	160	-2.213	-0.254	-2.486	**0.0139**	**0.0279**
**Nov. 24: Control - Apple**	-1.14	0.496	160	-2.124	-0.165	-2.307	**0.0224**	**0.0279**

Table S8. Statistical analyses of the *A. thailandicus* treatment. We manipulated the gut microbiome by supplementing apple food with *Acetobacter thailandicus*; the control in this experiment was apple food. (A) We ran a linear mixed effects model to determine how timepoint, treatment (control vs. *A. thailandicus*), and their interaction influenced pigmentation patterns. Cage was included in the model as a random effect. (B) Planned comparisons showing whether the control and *A. thailandicus* treatments exhibited significant shifts in pigmentation from summer to fall, and whether there were significant differences in pigmentation between treatments at the end of the summer (Sep. 7) and fall (Nov. 24) phases. Raw *p*-values and 95% confidence intervals are reported, as well as *p*-values following Holm correction for multiple comparisons.

A **Resident Microbe Additions: *At* (2020)**

Pigmentation Score ~ Timepoint * Treatment + (1 | Cage)

	Sum Sq	Mean Sq	Num DF	Den DF	F value	*p*-value
Timepoint	174.448	87.224	2	524	7.8460	**0.0004**
Treatment	4.859	4.859	1	10	0.4371	0.5235
Timepoint x Treatment	105.100	52.550	2	524	4.7270	**0.0092**

B Contrasts

Contrast	Estimate	SE	Df	Lower CL	Upper CL	*t* ratio	*p*-value	Adjusted *p*-value (Holm)
Control (Apple): Sep. 7 - Nov. 24	1.722	0.497	524	0.7458	2.70	3.465	**0.0006**	**0.0023**
At (Apple + *At*): Sep. 7 - Nov. 24	0.956	0.497	524	-0.0209	1.93	1.923	0.0551	0.1102
Sep. 7: Control - *At*	1.189	0.527	58	0.1341	2.24	2.256	**0.0278**	0.0835
Nov. 24: Control - *At*	0.422	0.527	58	-0.6326	1.48	0.801	0.4263	0.4263

Table S9. Statistical analyses of the *L. brevis* treatment. We manipulated the gut microbiome by supplementing apple food with *Lactobacillus brevis*; the control in this experiment was apple food. (A) We ran a linear mixed effects model to determine how timepoint, treatment (control vs. *L. brevis*), and their interaction influenced pigmentation patterns. Cage was included in the model as a random effect. (B) Planned comparisons showing whether the control and *L. brevis* treatments exhibited significant shifts in pigmentation from summer to fall, and whether there were significant differences in pigmentation between treatments at the end of the summer (Sep. 7) and fall (Nov. 24) phases. Raw *p*-values and 95% confidence intervals are reported, as well as *p*-values following Holm correction for multiple comparisons.

A Resident Microbe Additions: *Lb* (2020)

Pigmentation Score ~ Timepoint * Treatment + (1 | Cage)

	Sum Sq	Mean Sq	Num DF	Den DF	F value	*p*-value
Timepoint	70.415	35.207	2	524	3.4758	**0.0317**
Treatment	13.399	13.399	1	10	1.3227	0.2769
Timepoint x Treatment	169.615	84.807	2	524	8.3724	**0.0003**

B Contrasts

Contrast	Estimate	SE	Df	Lower CL	Upper CL	*t* ratio	*p*-value	Adjusted *p*-value (Holm)
Control (Apple): Sep. 7 - Nov. 24	1.722	0.474	524.0	0.790	2.654	3.630	**0.0003**	**0.0012**
Lb (Apple + *Lb*): Sep. 7 - Nov. 24	-0.656	0.474	524.0	-1.588	0.276	-1.382	0.1676	0.1676
Sep. 7: Control - *Lb*	1.244	0.488	69.5	0.272	2.217	2.552	**0.0129**	**0.0388**
Nov. 24: Control - *Lb*	-1.133	0.488	69.5	-2.106	-0.161	-2.324	**0.0231**	**0.0461**

Table S10. Monthly Climate Normals for Philadelphia, PA. To illustrate typical monthly shifts in temperature in Philadelphia, PA, USA, we utilized the most current dataset curated by the NOAA National Centers for Environmental Information (NOAA NCEI). This dataset is the "U.S. Monthly Climate Normals (2006-2020)", and it reports the monthly average of meteorological parameters recorded by weather stations across 15 years for sites across the United States. Here, we report the mean daily maximum, daily minimum, and overall daily temperature for Philadelphia, as recorded by the station "Philadelphia Franklin Institute, PA US USC00366886" (Palecki et al., 2021).

**Month**	**Mean Daily Maximum Temperature (°C)**	**Mean Daily Minimum Temperature (°C)**	**Mean Daily Temperature (°C)**
January	5.94	-2.22	1.89
February	7.11	-1.78	2.67
March	12.39	2.50	7.44
April	19.11	8.22	13.67
May	24.56	13.94	19.28
June	29.50	19.06	24.28
July	32.33	22.28	27.28
August	30.83	20.89	25.89
September	27.17	17.39	22.28
October	20.56	11.17	15.83
November	13.83	4.94	9.39
December	8.94	1.06	5.00

References

Palecki, M., Durre, I., Applequist, S., Arguez, A., Lawrimore, J. (2021). *U.S. Climate Normals 2020: U.S. Monthly Climate Normals (2006-2020).* [Dataset]. NOAA National Centers for Environmental Information. https://doi.org/10.25921/n5tw-jb91
